# Synergistic therapeutic effects of metformin and curcumin on polycystic ovary syndrome via regulation of insulin resistance and oxidative stress in a rat model

**DOI:** 10.3389/fendo.2026.1675883

**Published:** 2026-02-16

**Authors:** Lina Zheng, Baohua Wu, Junli Zhang, Yianduo Zhang, Zhiling Yang, Caifeng Deng, Chaolin Huang, Ling Lu

**Affiliations:** 1 Department of Gynecology and Obstetrics, The First Affiliated Hospital of Chengdu Medical College, Chengdu, China; 2 Department of Pathology, The First Affiliated Hospital of Chengdu Medical College, Chengdu, China

**Keywords:** curcumin, insulin resistance, metformin, oxidative stress, polycystic ovary syndrome

## Abstract

**Objective:**

Polycystic ovary syndrome (PCOS) is a common endocrine and metabolic disorder characterized by insulin resistance, hyperandrogenism, and elevated oxidative stress. The present study aimed to investigate the synergistic therapeutic effects of metformin and curcumin on insulin resistance and oxidative stress in a rat model of PCOS.

**Materials and methods:**

Thirty female Sprague-Dawley rats were randomly assigned to blank control (n = 6) and experimental (PCOS) groups (n = 24). The PCOS model was induced through daily subcutaneous injections of dehydroepiandrosterone (DHEA, 60 mg/kg) for 20 days. Subsequently, the experimental group was subdivided into four groups: PCOS-only, PCOS-metformin (300 mg/kg), PCOS-curcumin (50 mg/kg), and PCOS-metformin-curcumin (combined treatment). Treatments were administered daily via oral gavage for another 20 days. Estrous cycles, ovarian morphology, serum sex hormones, insulin resistance, and oxidative stress in ovarian tissues were assessed.

**Results:**

Combined treatment with metformin and curcumin significantly restored estrous cyclicity, improved ovarian morphology, reduced serum testosterone and luteinizing hormone levels, decreased LH/FSH ratio, and ameliorated insulin resistance compared to monotherapy groups. Moreover, the combined treatment markedly decreased ovarian oxidative stress markers (ROS and MDA) and enhanced antioxidant enzyme activities (GPx, SOD, GSH), surpassing the effects of either agent alone.

**Conclusion:**

Combined administration of metformin and curcumin demonstrated synergistic therapeutic effects in PCOS rats by concurrently addressing insulin resistance and oxidative stress. These findings support the clinical potential of this combination as an effective strategy for managing PCOS. Further clinical studies are necessary to validate these promising preclinical findings.

## 1 Introduction

Polycystic ovary syndrome (PCOS) is a prevalent endocrine and metabolic disorder affecting approximately 8-13% of women of reproductive age (1, 2). PCOS is characterized by irregular menstrual cycles, hyperandrogenism, insulin resistance, and polycystic ovaries (3, 4). It is also associated with various metabolic complications, including obesity, dyslipidemia, impaired glucose tolerance, and elevated oxidative stress (5–7). Despite the availability of several therapeutic strategies, current treatments such as lifestyle modification, oral contraceptives, and insulin sensitizers frequently exhibit limited efficacy and adverse side effects, underscoring an urgent need for safer and more effective therapeutic approaches (8).

Metformin is an oral hypoglycemic agent belonging to the biguanide class and is widely used to manage insulin resistance and metabolic abnormalities associated with PCOS (9, 10). It primarily reduces hepatic glucose production, enhances peripheral insulin sensitivity, and has demonstrated beneficial effects in restoring menstrual cyclicity and reducing androgen levels (11). Nevertheless, the clinical application of metformin is often constrained by gastrointestinal intolerance, including nausea, vomiting, and diarrhea (11–13). Furthermore, variable patient responses necessitate the exploration of adjunctive therapies to improve its efficacy and tolerability in the management of PCOS.

Curcumin is a bioactive polyphenolic component derived from turmeric, which has recently emerged as a promising candidate for PCOS treatment due to its anti-inflammatory, antioxidant, and insulin-sensitizing properties (14–16). Previous studies indicate that curcumin exerts beneficial effects in PCOS by modulating inflammatory pathways, reducing oxidative stress, and regulating insulin signaling cascades such as the PI3K/AKT/mTOR pathway (17). Specifically, curcumin has demonstrated the ability to improve ovarian morphology, restore estrous cycles, decrease hyperandrogenism, and alleviate insulin resistance in animal models of PCOS (16). Despite these encouraging preclinical findings, clinical data supporting the therapeutic efficacy of curcumin in PCOS remain limited, prompting further investigation to validate its clinical potential.

The combination of metformin and curcumin represents an innovative therapeutic strategy, potentially capitalizing on their complementary mechanisms to yield synergistic benefits. The glucose-regulating and insulin-sensitizing effects of metformin, coupled with the antioxidative and anti-inflammatory actions of curcumin, may collectively address both metabolic and reproductive abnormalities associated with PCOS. Thus, the present study aims to investigate the synergistic effects of metformin and curcumin on insulin resistance and oxidative stress in a rat model of PCOS. Findings from this study may provide critical insights into the combined clinical applicability and significance of these agents in enhancing therapeutic outcomes for patients with PCOS.

## 2 Materials and methods

### 2.1 Experimental animals and treatments

The experimental procedures were reviewed and authorized by the Ethics Committee of Chengdu Medical College (Approval Number: 24341325). A total of 30 Sprague-Dawley (SD) rats, approximately 6 weeks old and weighing around 168 ± 7 g, were housed in our institutional animal facility under controlled environmental conditions (temperature maintained at 22 ± 2 °C and a 12-hour cycle of alternating light and darkness). Food and water were provided freely. After a one-week acclimatization, the 30 female rats were randomized to a blank control group (n=6) and an experimental group (PCOS) (n=24) using a computer-generated sequence.

To create a PCOS rat model, the experimental group received daily subcutaneous injections containing 60 mg/kg body weight of dehydroepiandrosterone (DHEA), sourced from Sigma-Aldrich (Darmstadt, Germany), dissolved in 100 mL of sesame oil. These treatments continued for 20 consecutive days according to previously established methods and the manufacturer’s guidelines (18). Successful induction of the PCOS rat model was verified using standard diagnostic criteria (19).

Following successful PCOS induction, experimental rats were re-randomized in permuted blocks (block size = 6) to four subgroups (PCOS-only, PCOS-metformin, PCOS-curcumin, PCOS-metformin-curcumin; n=6 each) to maintain balance. Group allocation was balanced across cages (3 rats/cage) to minimize potential cage effects. Allocation lists were prepared by a technician independent of outcome assessment; group codes were kept in sealed envelopes, and dosing bottles were labeled only with anonymous codes.

Rats in the PCOS-metformin subgroup were administered metformin (300 mg/kg body weight; Product No. 317240, Sigma-Aldrich) daily via oral gavage (20, 21). Those in the PCOS-curcumin subgroup received curcumin (50 mg/kg body weight; Product No. PHR2209, Sigma-Aldrich) daily through oral gavage. The combined treatment subgroup (PCOS-metformin-curcumin) was simultaneously administered metformin and curcumin at identical dosages as the individual treatment subgroups. The PCOS-only subgroup was given 200 mL of distilled water daily via oral gavage.

### 2.2 Assessment of estrous cycles of experimental rats

Every day at 09:00 AM, vaginal smears were taken and spread evenly on glass slides, air-dried, and subsequently stained using 0.1% methylene blue solution. Microscopic examinations were performed, and representative images were recorded. Estrous cycle phases were classified based on vaginal cytology observations (**Supplementary Figures 6A–D**). The proestrus stage was indicated by the prevalence of nucleated epithelial cells; the estrus stage was recognized by abundant cornified squamous epithelial cells; the metestrus stage featured a mixture of cornified squamous epithelial cells and leukocytes; and the diestrus stage was defined by a predominance of leukocytes (19).

### 2.3 Assessment of sex hormones

To minimize variations in sex hormone levels caused by different phases of the estrous cycle, blood was drawn from the caudal veins of rats specifically during the diestrus stage. The collected blood was centrifuged at 2000 × g for 10 minutes to isolate the serum. Concentrations of luteinizing hormone (LH,MB-3750B, Meibiao, Jiangsu, China), testosterone (T, MB-2118B, Meibiao, Jiangsu, China),follicle-stimulating hormone (FSH, MB-7343B, Meibiao, Jiangsu, China), estradiol (E2, MB-2116B, Meibiao, Jiangsu, China), progesterone (MB-6832B, Meibiao, Jiangsu, China), prolactin (MB-2104B, Meibiao, Jiangsu, China), and anti-Müllerian hormone (AMH, MB-6799B, Meibiao, Jiangsu, China)were determined using enzyme-linked immunosorbent assay (ELISA)kits, following the protocols provided by the manufacturer. All serum samples were analyzed in triplicate to ensure accuracy.

### 2.4 Assessment of insulin resistance

The rats fasted for 12 hours before collecting the blood for measurement of fasting insulin(MB-2128B, Meibiao, Jiangsu, China) and fasting blood glucose(590, Yuwell, Jiangsu, China). The levels of fasting insulin and fasting blood glucose were determined using commercial assay kits, following the protocols provided by the manufacturer. Insulin resistance index (HOMA-IR) was calculated by the formula of fasting insulin (μU/mL) × fasting blood glucose (mmol/L)/22.5 as described in the previously published study (22).

### 2.5 Assessment of oxidative stress

Oxidative Stress in ovarian tissues was assessed by commercial assay kits. The oxidative stress markers, specifically reactive oxygen species (ROS) and malondialdehyde (MDA), were quantified using commercial assay kits, including the ROS Detection Assay Kit (ab186027, Abcam, Shanghai, China) and the Lipid Peroxidation (MDA) Assay Kit (ab233471, Abcam), respectively. Additionally, antioxidant activity markers such as superoxide dismutase (SOD), glutathione peroxidase (GPx), and glutathione (GSH) were measured utilizing assay kits including the Superoxide Dismutase Activity Assay Kit (ab65354, Abcam) (39), Glutathione Peroxidase Assay Kit (ab102530, Abcam) (14), and Glutathione Assay Kit (ab65322, Abcam) (14), respectively. All procedures followed the manufacturer-provided protocols.

### 2.6 Ovarian morphological analysis

We euthanized the experimental rats by intraperitoneal injection with sodium pentobarbital at a dose of 150 mg/kg. The ovaries of the rats were extracted, immersed in 4% paraformaldehyde solution, and maintained at 4 °C overnight for fixation. The tissue samples were then embedded in paraffin wax. Subsequently, serial sections of 4 μm thickness were cut from these paraffin-embedded samples and stained with hematoxylin and eosin (H&E) according to conventional histological procedures. Ovarian morphology and the quantity of follicular cysts were assessed by microscopic examination(40.82x,BX51,Olympus).

### 2.7 Statistical analysis

The study was prospectively powered for the endpoint of insulin resistance index, comparing the PCOS group with the metformin and curcumin combination group using a two-sided independent t-test (α = 0.05). Pilot data from our laboratory (same rat strain and assays) indicated an expected between-group difference in insulin resistance index of 0.2 units with a pooled standard deviation of 0.14 (Cohen’s d=1.62). The sample size for each group was calculated by the standard formula n=2 (Z_1−α/2_+Z_1−β_)^2^/d^2^, with 95% power, Z_1−β_ = 0.84, and Z_1−α/2_ =1.96; the required sample size was calculated to be 5.9 per group. We therefore enrolled n = 6 per group (five groups; total N = 30) to ensure ≥95% power while accommodating potential attrition.

Data analysis was conducted using GraphPad Prism 6.01. Comparisons between two groups were made using an unpaired Student’s t-test. For studies with three or more groups, use Brown-Forsythe and Welch ANOVA tests, followed by Dunnett T3 tests to. A p-value of less than 0.05 was regarded as indicating statistical significance.

## 3 Results

### 3.1 DHEA induced the PCOS model in rats

**Figure 1:**
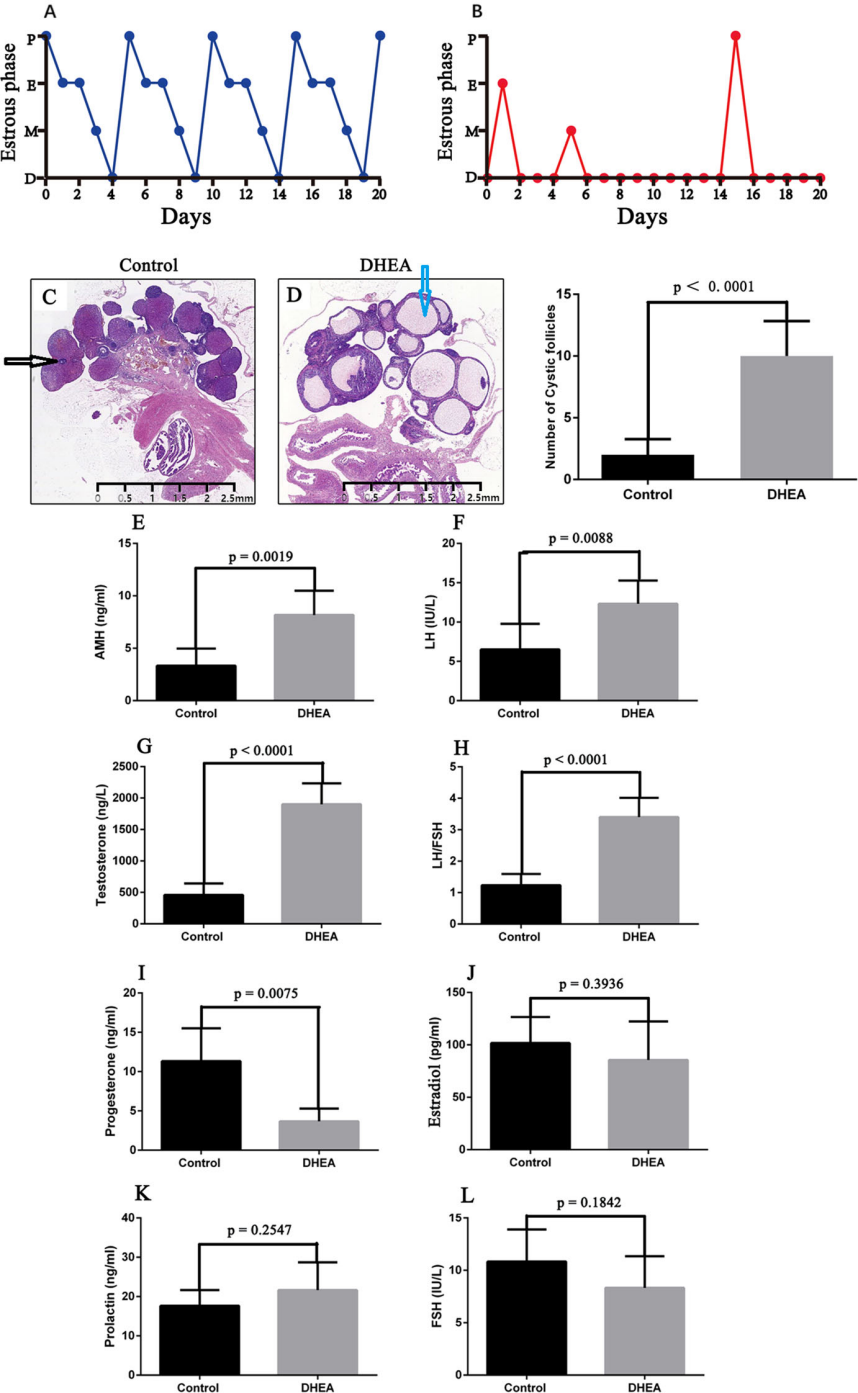
DHEA induced the PCOS model in rats. **(A)** Normal sequential estrous cycles in the control rat group, progressing through proestrus, estrus, metestrus, and diestrus phases. **(B)** DHEA-treated rats exhibited irregular estrous cycles, with most cycles being disrupted, resulting in a prolonged stay in the diestrus phase. **(C)** Normal ovarian structure in the control rat group. **(D)** The ovaries of DHEA-treated rats had an increased number of follicles exhibiting cystic enlargement. The blue arrows indicate cystic follicles, and the black arrows indicate follicles **(C, D)**. **(E-I)** DHEA-treated rats exhibited significantly higher concentrations of anti-Müllerian hormone **(E)**, luteinizing hormone **(F)**, and testosterone **(G)**, along with an elevated LH/FSH ratio **(H)**. Progesterone levels were significantly reduced in rats treated with DHEA **(I)**. **(J–L)** DHEA treatment didn’t change the levels of estradiol **(J)**, prolactin **(K)**, and follicle-stimulating hormone **(L)**. Comparisons between two groups were made using an unpaired Student’s t-test. A p-value of less than 0.05 was regarded as indicating statistical significance. P, proestrus; E, estrus; M, metestrus; D, diestrus; DHEA, dehydroepiandrosterone; AMH, anti-Müllerian hormone; LH, luteinizing hormone; FSH, follicle-stimulating hormone.

Rats in the control group displayed consistent and sequential estrous cycles, progressing through proestrus, estrus, metestrus, and diestrus phases (**Figure 1A**). In contrast, rats treated with DHEA showed irregular estrous cycles that were mostly disrupted, with a prolonged stay in the diestrus phase (**Figure 1B**). Histological analysis revealed that the control group maintained normal ovarian structure, while the ovaries of DHEA-treated rats had an increased number of follicles exhibiting cystic enlargement (**Figures 1C, D**).

Furthermore, serum sex hormone levels were evaluated. Relative to controls, rats receiving DHEA had significantly higher concentrations of anti-Müllerian hormone (AMH) (p = 0.0019), luteinizing hormone (LH) (p = 0.0088), and testosterone (p < 0.0001), along with an elevated LH/FSH ratio (p < 0.0001) when compared to control rats (**Figures 1E-H**). Conversely, progesterone levels were significantly reduced in DHEA-treated rats (p = 0.0075) (**Figure 1I**). There was no statistically significant difference in estradiol (p = 0.3936), prolactin (p = 0.2547), and follicle-stimulating hormone (FSH) (p = 0.1842) levels between the two groups (**Figures 1J-L**). These results confirmed the successful establishment of the PCOS model in rats (18).

### 3.2 Effects of metformin and curcumin on sex hormone dysregulation in PCOS rats

We investigated the effects of metformin and curcumin on sex hormone dysregulation in a PCOS rat model. We used LC-MS/MS to assess the plasma drug levels of metformin and curcumin in the combination group versus the monotherapy groups and found that combination group didn’t increase plasma levels of either the two drugs.

**Figure 2:**
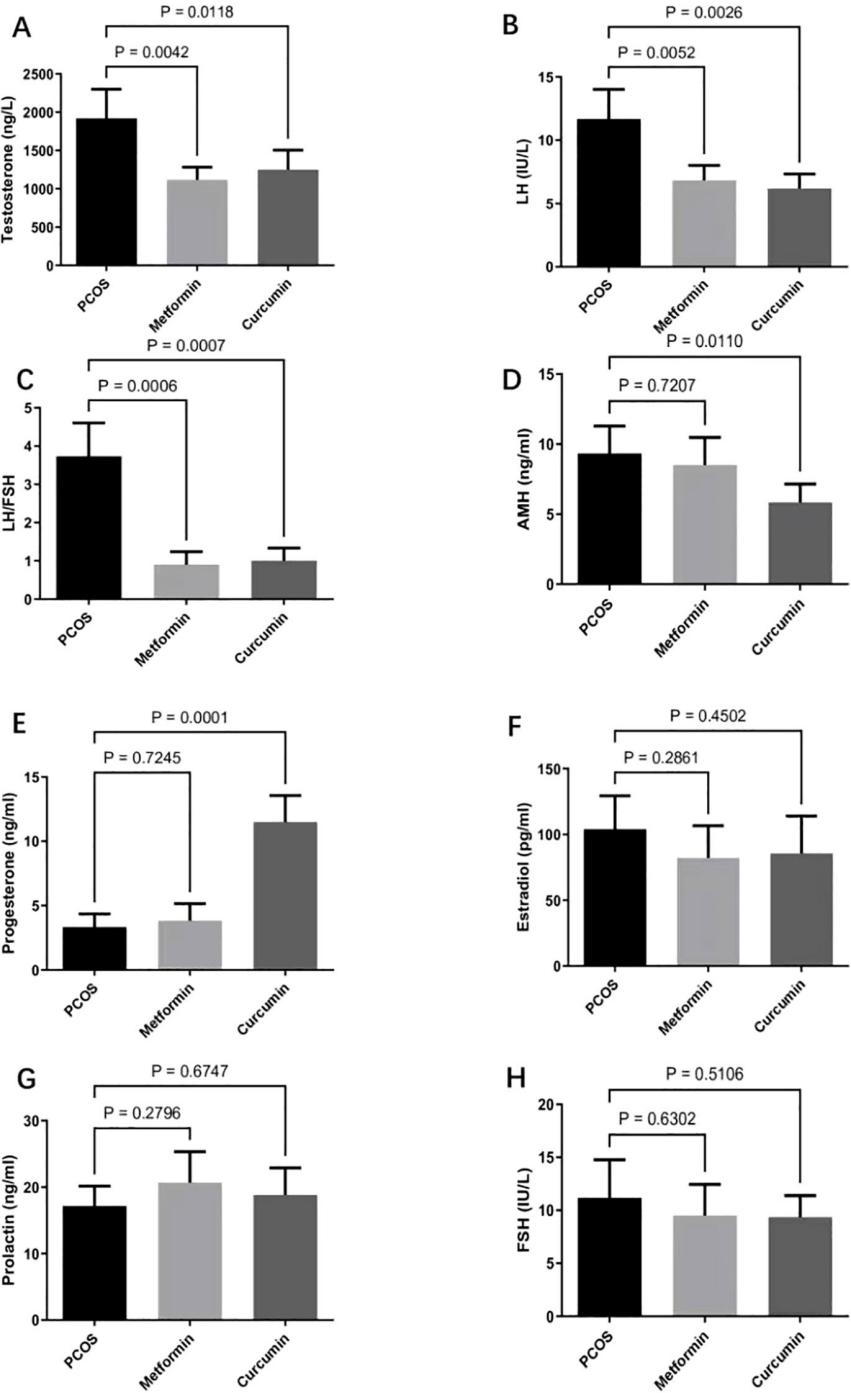
Effects of metformin and curcumin on sex hormone dysregulation in PCOS rats. **(A–C)** Metformin significantly decreased serum levels of testosterone **(A)**, as well as lowered the LH/FSH ratio **(C)**. **(D–H)** Metformin had no significant effect on luteinizing hormone **(B)**, AMH **(D)**, progesterone **(E)**, estradiol **(F)**, prolactin **(G)**, and FSH **(H)**. Curcumin significantly reduced levels of testosterone **(A)**, luteinizing hormone **(B)**, and AMH **(D)**, and lowered the LH/FSH ratio **(C)**. Curcumin significantly increased levels of progesterone **(E)** but did not affect estradiol **(F)**, prolactin **(G)**, and FSH **(H)**. For studies with three or more groups, use Brown-Forsythe and Welch ANOVA tests, followed by Dunnett T3 tests to. A p-value of less than 0.05 was regarded as indicating statistical significance. AMH, anti-Müllerian hormone; LH, luteinizing hormone; FSH, follicle-stimulating hormone.

Metformin significantly decreased serum levels of testosterone (p = 0.0042) and luteinizing hormone (p = 0.0052), as well as lowered the LH/FSH ratio (p=0.0006), compared with PCOS rats (**Figures 2A–C**). However, metformin had no significant effect on AMH (p = 0.7207), progesterone (p = 0.7245), estradiol (p = 0.2861), prolactin (p = 0.2796), or FSH (p = 0.6302) (**Figures 2D–H**). Curcumin significantly reduced serum levels of testosterone (p = 0.0118), luteinizing hormone (p = 0.0026), and AMH (p = 0.0110), and also lowered the LH/FSH ratio (p=0.0007) compared with controls (**Figures 2A–D**). Conversely, curcumin significantly increased serum levels of progesterone compared with PCOS rats (p=0.0001) (**Figure 2E**). However, curcumin did not significantly affect the levels of estradiol (p = 0.4502), prolactin (p = 0.6747), and FSH (p = 0.5106) (**Figures 2F–H**).

### 3.3 Effects of metformin and curcumin on the estrous cycle and ovarian morphology in PCOS rats

**Figure 3:**
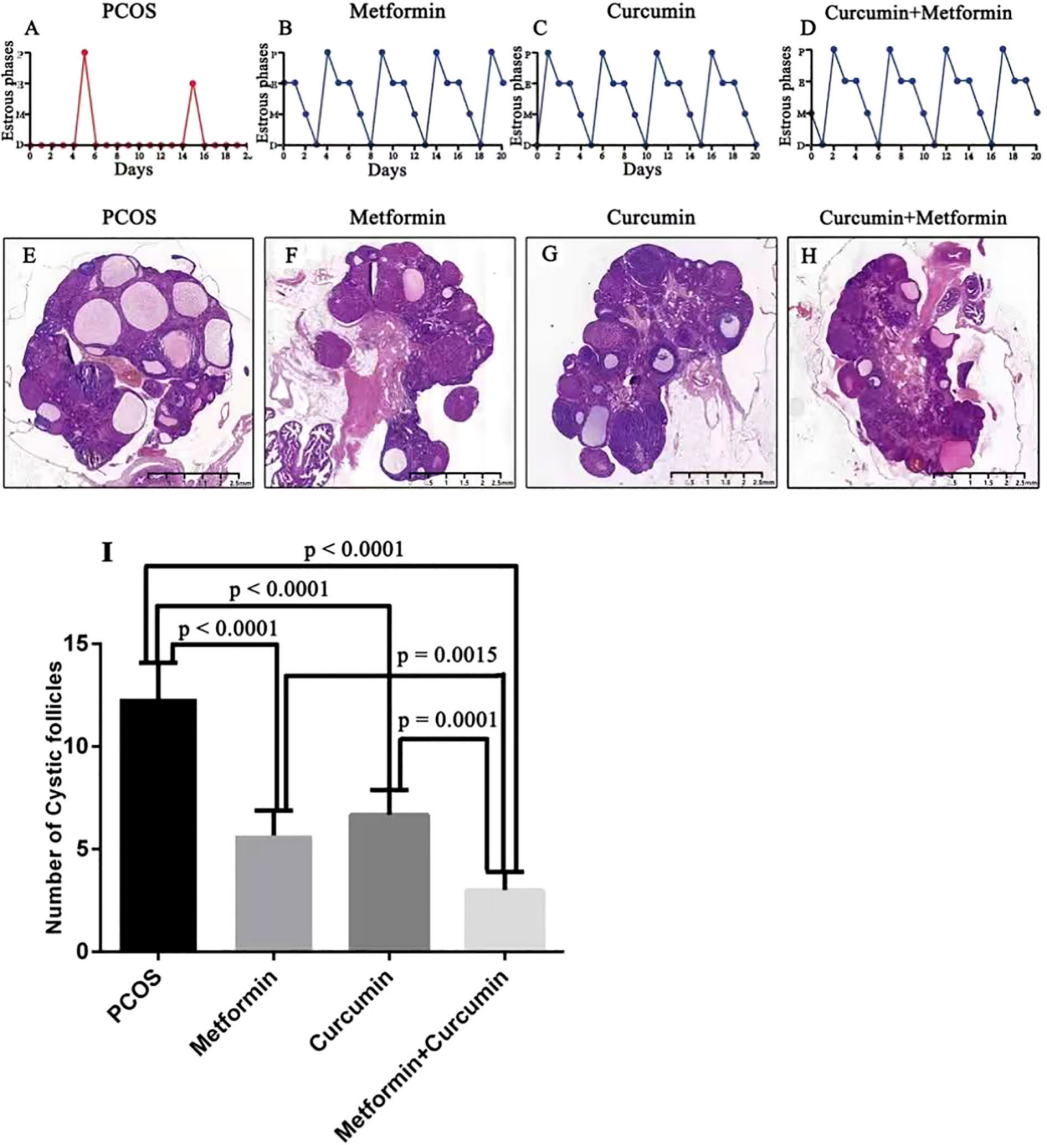
Effects of metformin and curcumin on estrous cycle and ovarian morphology in PCOS rats. **(A)** The estrous cycles in PCOS rats were irregular, with the animals predominantly remaining in the diestrus phase. **(B-D)** Metformin and curcumin helped restore normal estrous cycling in PCOS rats, the blue arrows indicate cystic follicles, and the black arrows indicate follicles. **(E)** Ovarian tissue from PCOS rats exhibited typical polycystic alterations. **(F–H)** Metformin and curcumin helped restore the normal ovarian morphology, which contained multiple corpora lutea, indicating resumed ovulation. **(I)** The number of cystic follicles was significantly reduced in the metformin and curcumin-treated PCOS rats. **(I)** The combined use of metformin and curcumin further reduced the number of cystic follicles. For studies with three or more groups, use Brown-Forsythe and Welch ANOVA tests, followed by Dunnett T3 tests to. A p-value of less than 0.05 was regarded as indicating statistical significance. P, proestrus; E, estrus; M, metestrus; D, diestrus.

The estrous cycles in PCOS rats were irregular, with the animals predominantly remaining in the diestrus phase (**Figure 3A**). Treatment with metformin and curcumin helped restore normal estrous cycling in these rats (**Figures 3B–D**). Moreover, ovarian tissue from PCOS rats exhibited typical polycystic alterations (**Figure 3E**). Following administration of metformin and curcumin, the ovaries no longer displayed polycystic features but contained multiple corpora lutea, indicating resumed ovulation (**Figures 3F–H**). Additionally, the number of cystic follicles was significantly reduced in the metformin (p < 0.0001) and curcumin-treated (p < 0.0001) PCOS rats (**Figure 3I**). Most importantly, the combined use of metformin and curcumin further reduced the number of cystic follicles (p = 0.0015 and p = 0.0001) (**Figure 3I**).

### 3.4 Effects of metformin and curcumin on insulin resistance in PCOS rats

**Figure 4:**
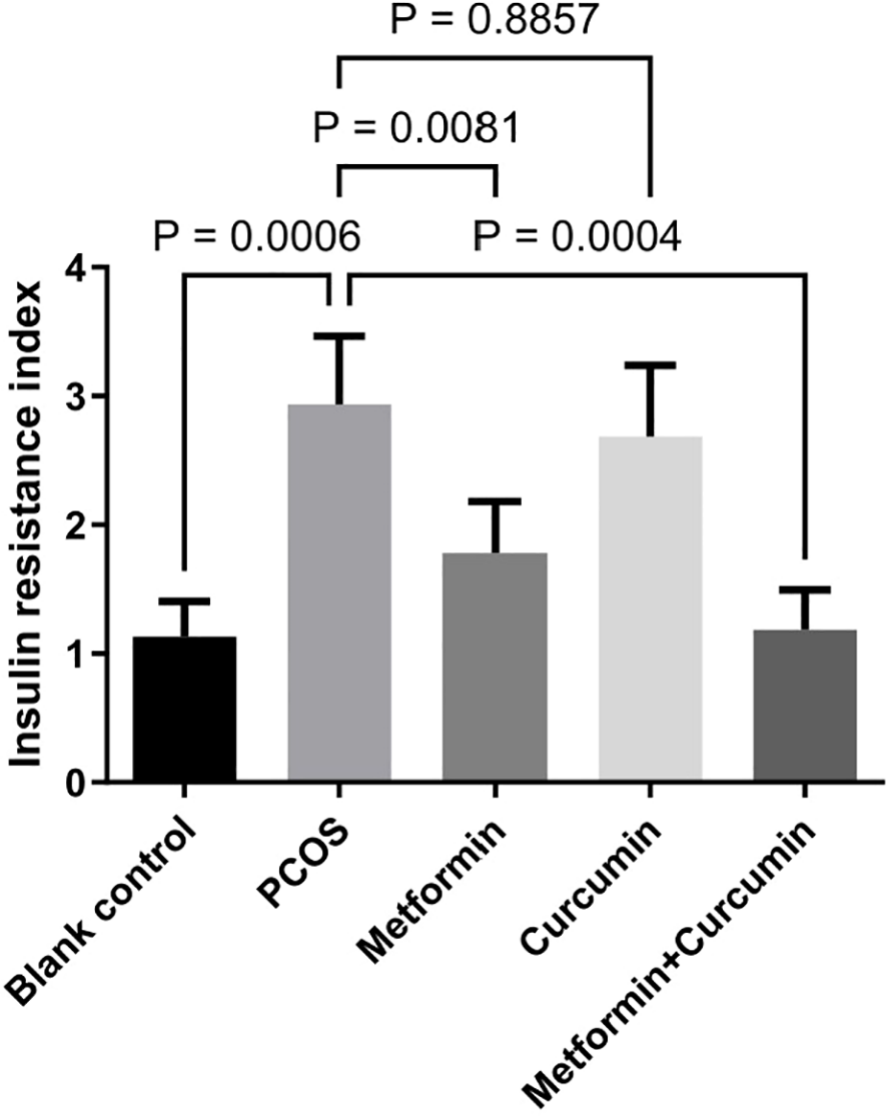
Effects of metformin and curcumin on insulin resistance in PCOS rats. Metformin treatment effectively reduced insulin resistance in PCOS rats (p = 0.0017). Curcumin alone did not produce a significant alleviation in insulin resistance (p = 0.4447). The combination of metformin and curcumin exerted a more pronounced effect on reducing insulin resistance compared to metformin treatment alone (p = 0.0156). For studies with three or more groups, use Brown-Forsythe and Welch ANOVA tests, followed by Dunnett T3 tests to. A p-value of less than 0.05 was regarded as indicating statistical significance.

PCOS rats exhibited a significantly elevated HOMA-IR index, indicating the presence of insulin resistance (p=0.0006) (**Figure 4**). Metformin treatment effectively reduced insulin resistance in these rats (p = 0.0081). In contrast, curcumin alone did not produce a significant alleviation in insulin resistance (p = 0.8857) (**Figure 4**). However, the combination of metformin and curcumin exerted a more pronounced effect on reducing insulin resistance compared to metformin treatment alone (p = 0.0004) (**Figure 4**).

### 3.5 Effects of metformin and curcumin on oxidative stress in PCOS rats

**Figure 5:**
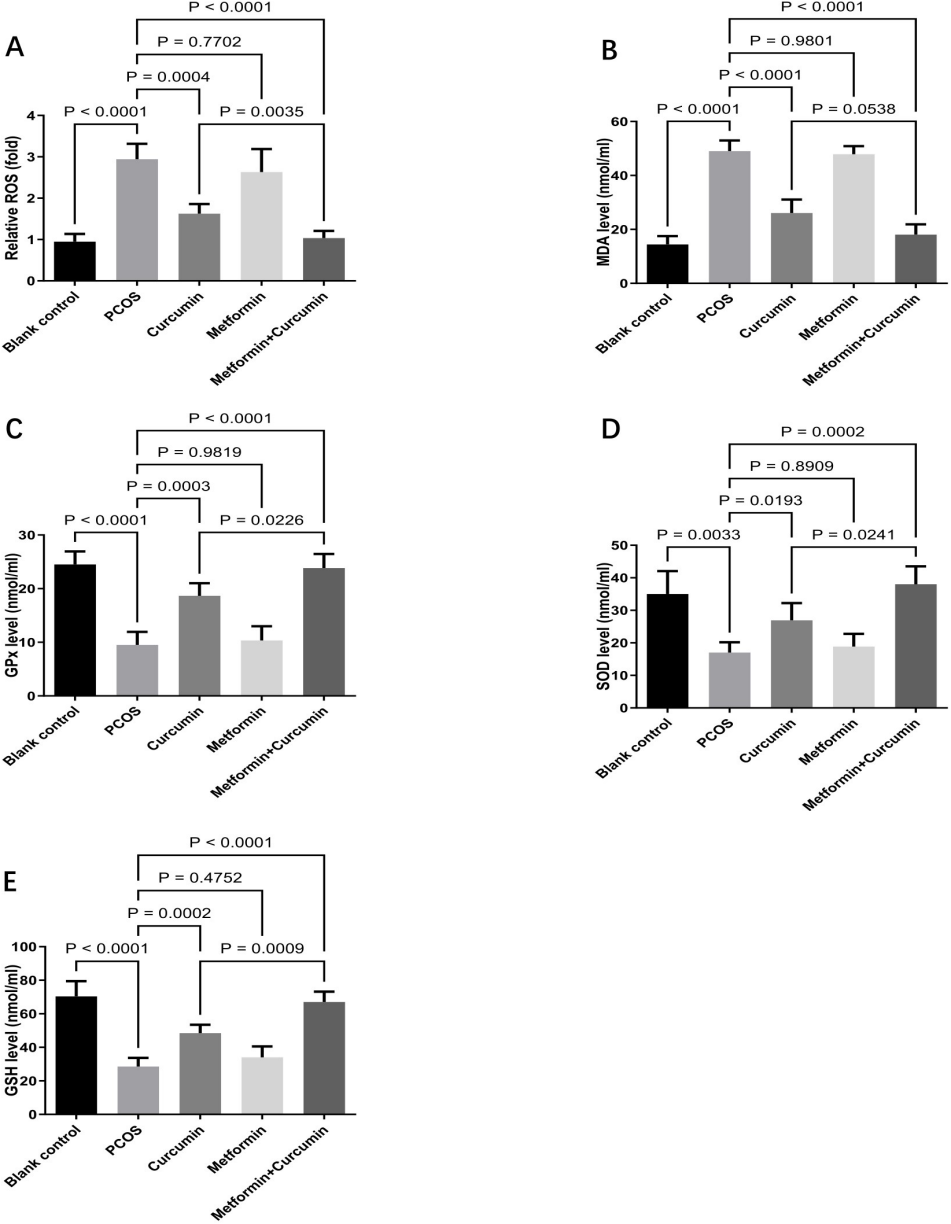
Effects of metformin and curcumin on oxidative stress in PCOS rats. **(A)** Effects of metformin and curcumin on reactive oxygen species. **(B)** Effects of metformin and curcumin on malondialdehyde. **(C)** Effects of metformin and curcumin on glutathione peroxidase. **(D)** Effects of metformin and curcumin on superoxide dismutase. **(E)** Effects of metformin and curcumin on glutathione. For studies with three or more groups, use Brown-Forsythe and Welch ANOVA tests, followed by Dunnett T3 tests to. A p-value of less than 0.05 was regarded as indicating statistical significance. GSH, glutathione; GPx, glutathione peroxidase; MDA, malondialdehyde; ROS, reactive oxygen species; SOD, superoxide dismutase.

Oxidative stress within the ovaries of PCOS rats was assessed by measuring reactive oxygen species and key enzymes involved in redox regulation. Findings indicated that PCOS rats had markedly elevated oxidative markers, including reactive oxygen species (p < 0.0001) and malondialdehyde (p < 0.0001), coupled with reduced antioxidant enzyme levels such as glutathione peroxidase (p < 0.0001), superoxide dismutase (p=0.0033), and glutathione (p < 0.0001) in ovarian tissue (**Figures 5A–E**). Treatment with curcumin significantly lowered the levels of reactive oxygen species (p =0.0004) and malondialdehyde (p < 0.0001) while increasing the levels of glutathione peroxidase (p=0.0003), superoxide dismutase (p = 0.0193),and glutathione (p=0.0002) in the ovaries (**Figures 5A–E**). In contrast, metformin alone did not significantly alter these oxidative stress indicators (all p > 0.05). However, the combined administration of curcumin and metformin produced a stronger reduction in oxidative stress than curcumin treatment alone (**Figures 5A–E**).

## 4 Discussion

In the present study, we demonstrated that the combined administration of metformin and curcumin exerted synergistic therapeutic effects on PCOS in a rat model, primarily by regulating insulin resistance and oxidative stress. Our findings revealed that the combination treatment effectively normalized estrous cyclicity, improved ovarian morphology, significantly reduced serum testosterone and luteinizing hormone levels, decreased the LH/FSH ratio, and alleviated insulin resistance. Additionally, curcumin, particularly when combined with metformin, markedly reduced oxidative stress in the ovarian tissues of PCOS rats. These results highlight the clinical potential of combining metformin and curcumin as a safer and more effective therapeutic approach for PCOS management compared to metformin monotherapy.

Metformin, significantly improves glucose metabolism by inhibiting hepatic gluconeogenesis and enhancing peripheral glucose uptake (23, 24). Despite its broad clinical use and proven efficacy in improving insulin sensitivity, metformin alone often fails to adequately address other critical aspects of PCOS pathophysiology, notably oxidative stress and chronic ovarian inflammation (25, 26). Increased oxidative stress, characterized by elevated production of ROS and reduced antioxidant defense, substantially contributes to ovarian dysfunction and impaired follicular development in PCOS (27, 28). Furthermore, chronic inflammation in PCOS exacerbates ovarian and systemic insulin resistance, thus accelerating disease progression (29). In addition, metformin therapy is frequently accompanied by gastrointestinal side effects such as nausea, diarrhea, and bloating, leading to compromised patient adherence and therapeutic effectiveness (13). Consequently, adjunctive therapies simultaneously targeting oxidative stress and inflammatory pathways represent a rational and promising therapeutic advancement in PCOS treatment.

Curcumin, has gained attention as a promising adjunctive therapeutic candidate for PCOS due to its potent anti-inflammatory and antioxidant effects (30, 31). Previous animal studies have reported that curcumin can enhance ovarian function and fertility by alleviating oxidative stress and chronic inflammation within ovarian tissues (14, 32, 33). Nonetheless, clinical evidence supporting curcumin’s effectiveness in managing PCOS remains limited, emphasizing the need for further research to validate its therapeutic efficacy.

Considering the individual limitations of metformin and the promising therapeutic effects of curcumin by alleviating oxidative stress and chronic inflammation, we hypothesized that their combined application could yield superior outcomes in PCOS management. Indeed, our findings demonstrated significant synergistic effects of this combined treatment in terms of improving insulin resistance, regulating hormonal imbalances, and reducing oxidative stress. Notably, although curcumin alone did not significantly influence insulin sensitivity, it markedly enhanced the insulin-sensitizing effects of metformin. Furthermore, curcumin significantly reduced ovarian oxidative stress, which was not observed with metformin treatment alone. These complementary effects potentially explain the notable improvements in ovarian morphology and restoration of estrous cyclicity observed in rats treated with the combination therapy. These findings suggest that curcumin’s antioxidant and anti-inflammatory properties effectively enhanced the therapeutic effects of metformin on PCOS.

Our findings align with previous studies that have demonstrated beneficial outcomes from combined metformin and curcumin treatment on metabolic parameters, oxidative stress, and inflammatory responses across various metabolic disorders. For instance, Roxo et al. reported that the combined administration of curcumin and metformin significantly improved lipid profiles, reduced oxidative stress markers, and enhanced antioxidant enzyme activities compared to individual treatments in diabetic rat models (34). Similarly, Cao et al. observed that the combined use of curcumin and metformin effectively mitigated inflammation and oxidative stress-induced nephrotoxicity, highlighting their synergistic antioxidant and anti-inflammatory potentials (35). Collectively, these studies reinforce the hypothesis that combining curcumin with metformin could achieve superior therapeutic outcomes, particularly by attenuating oxidative stress and inflammatory pathways closely associated with PCOS pathogenesis. Hence, our findings provide robust preclinical evidence supporting the combined use of metformin and curcumin as a potential innovative therapeutic approach for PCOS.

Our study observed the synergistic therapeutic effects of metformin and curcumin on polycystic ovary syndrome via regulation of insulin resistance and oxidative stress may arise from pharmacodynamic synergy at the tissue level, not from improved pharmacokinetic. This effect was not additive but synergistic through molecular signaling interactions, because we found that combination treatment didn’t increase plasma levels of either the two drugs.

Previous study report that metformin activates AMPK, which suppresses NF-κB signaling and can promote Nrf2-dependent antioxidant responses (23). On the other hand, curcumin activates Nrf2 and inhibits NF-κB, amplifying antioxidant and anti-inflammatory programs (36). The literature also describes functional crosstalk between AMPK and Nrf2, providing a mechanistic basis for synergistic effects on redox homeostasis (37). Therefore, metformin’s AMPK-mediated suppression of inflammatory signaling can potentiate curcumin’s NF-κB inhibition and Nrf2 activation, rationalizing the combined suppression of oxidative stress and inflammation as we observed.

Previous study also report that curcumin activates AMPK and PPARγ while down-regulating NF-κB in metabolic tissues, improving insulin sensitivity (38). In PCOS rodent models, curcumin also restores PI3K/AKT/mTOR signaling and alleviates insulin resistance and inflammation (17). Metformin activates AMPK and improves hepatic gluconeogenesis and insulin sensitivity, while AMPK signaling interacts with PI3K/AKT/mTOR and down-regulates NF-κB signaling (23). Our study also observed that curcumin improved the insulin sensitivity of metformin. Therefore, that signaling crosstalk creates pharmacodynamic synergy at the tissue level to alleviate insulin resistance.

Despite the promising results observed, this study has several limitations. First, Although the DHEA-induced rat model of PCOS is widely used in research, it must be recognized that no animal model can fully replicate the complexity of human PCOS. For instance, the induced phenotype often reverses rapidly within weeks after discontinuation of treatment and fails to persist. Additionally, the DHEA model can only recapitulate certain features of the metabolic syndrome frequently associated with PCOS in patients. Secondly, our findings were derived from an animal model, and the therapeutic doses of metformin may be different in humans and rats. Therefore, direct translation to clinical practice warrants careful consideration and validation through clinical trials. Thirdly, the dose–response effects and the precise molecular mechanisms underlying the synergistic interaction between curcumin and metformin remain incompletely understood and require further exploration in future studies. Fourthly, the relatively low bioavailability of curcumin remains a major challenge for clinical translation, highlighted the need for future research using advanced formulations to enhance absorption and clinical applicability. Lastly, although oxidative stress markers and insulin resistance indices were comprehensively evaluated, the effects of the combination therapy on other inflammatory markers and molecular signaling pathways were not extensively examined, representing an important area for future investigation.

## 5 Conclusion

Our study provides evidence that combined treatment with metformin and curcumin exerts synergistic therapeutic effects on PCOS by concurrently regulating insulin resistance and oxidative stress. The combination therapy addresses the limitations associated with metformin monotherapy, especially in reducing oxidative stress and enhancing insulin sensitivity. This approach may represent a strategy for improving clinical outcomes and patient adherence in PCOS management. However, further clinical trials and detailed mechanistic studies are essential to validate these findings and fully elucidate the underlying molecular mechanisms involved.

## Data Availability

The original contributions presented in the study are included in the article/**Supplementary Material**. Further inquiries can be directed to the corresponding authors.

## Supplementary material

Supplementary Figure 6 Estrous cycle phases were classified based on vaginal cytology observations. The proestrus stage was indicated by the prevalence of nucleated epithelial cells **(A)**; the estrus stage was recognized by abundant cornified squamous epithelial cells **(B)**; the metestrus stage featured a mixture of cornified squamous epithelial cells and leukocytes **(C)**; and the diestrus stage was defined by a predominance of leukocytes **(D)**.
