# Supplementary figures and images for: Synergistic therapeutic effects of metformin and curcumin on polycystic ovary syndrome via regulation of insulin resistance and oxidative stress in a rat model
Source: Front Endocrinol (Lausanne). 2026 Feb 16;17:1675883. doi: 10.3389/fendo.2026.1675883 (PMC12950547; doi:10.3389/fendo.2026.1675883)

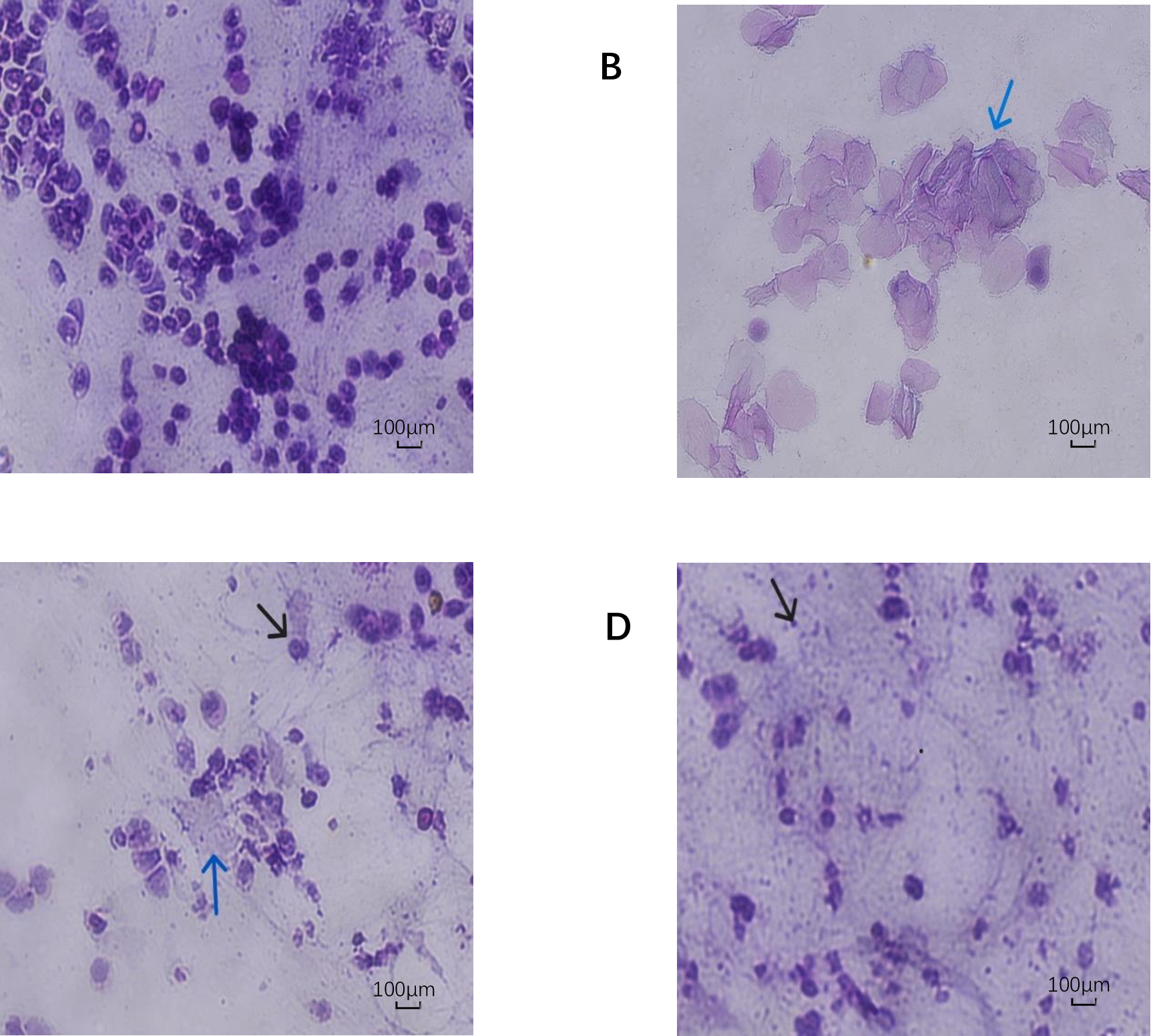

Supplement: Supplementary Figure 6 — Estrous cycle phases were classified based on vaginal cytology observations. The proestrus stage was indicated by the prevalence of nucleated epithelial cells (A); the estrus stage was recognized by abundant cornified squamous epithelial cells (B); the metestrus stage featured a mixture of cornified squamous epithelial cells and leukocytes (C); and the diestrus stage was defined by a predominance of leukocytes (D). [file Supplementaryfile1.tiff]
